# Clinical Outcomes with Alternative Dosing Strategies for Piperacillin/Tazobactam: A Systematic Review and Meta-Analysis

**DOI:** 10.1371/journal.pone.0116769

**Published:** 2015-01-09

**Authors:** Hui Yang, Chao Zhang, Quanyu Zhou, Yike Wang, Lujia Chen

**Affiliations:** 1 Department of pharmacy, Peking University Third Hospital, Beijing, China; 2 School of pharmaceutical sciences, Peking University, Beijing, China; 3 Department of pharmacy, The First People’s Hospital of Lianyungang, Jiangsu, China; University of Ottawa, CANADA

## Abstract

**Objectives:**

A better dosing strategy can improve clinical outcomes for patients. We sought to compare the extended or continuous infusion with conventional intermittent infusion of piperacillin/tazobactam, investigating which approach is better and worthy of recommendation for clinical use.

**Methods:**

Articles were gathered from PubMed, Web of Science, ProQuest, Science Direct, Cochrane, two Chinese literature databases (CNKI, Wan Fang Data) and related ICAAC and ACCP conferences. Randomized controlled and observational studies that compared extended or continuous infusion with conventional intermittent infusion of piperacillin/tazobactam were identified from the databases above and analyzed. Two reviewers independently extracted and investigated the data. A meta-analysis was performed using Revman 5.2 software. The quality of each study was assessed. Sensitivity analysis and publication bias were evaluated.

**Results:**

Five randomized controlled trials and nine observational studies were included in this study. All included studies had high quality and no publication bias was found. Compared to the conventional intermittent infusion approach, the extended or continuous infusion group had a significantly higher clinical cure rate (OR 1.88, 95% CI 1.29-2.73, P = 0.0009) and a lower mortality rate (OR 0.67, 95% CI 0.50-0.89, P = 0.005). No statistical difference was observed for bacteriologic cure (OR 1.40, 95% CI 0.82-2.37, P = 0.22) between the two dosing regimens. The sensitivity analysis showed the results were stable.

**Conclusions:**

Our systematic review and meta-analysis suggested that the extended or continuous infusion strategy of piperacillin/tazobactam should be recommended for clinical use considering its higher clinical cure rate and lower mortality rate in comparison with conventional intermittent strategy. Data from this study could be extrapolated for other β-lactam antimicrobials. Therefore, this dosing strategy could be considered in clinical practice.

## Introduction

Piperacillin/tazobactam is an extended-spectrum β-lactamase inhibitor combination antibiotic. Because of its broad coverage, piperacillin/tazobactam is commonly recommended as a first-line therapy for severe bacterial infections including intra-abdominal infection, hospital-acquired pneumonia, febrile neutropenia, and skin or soft-tissue infection [1], [2]. As a time-dependent antibiotic, the bactericidal activity of piperacillin/tazobactam is optimized when drug concentrations exceed the fractional time above the minimum inhibitory concentration (fT>MIC) for at least 30% to 50% [3–5].

As more is understood about antimicrobial agents through research, it is evidenced that proper use of antimicrobials can improve clinical outcomes and reduce resistance, while maintaining antimicrobial sensitivity in general population [6–10]. Conventional dosing of piperacillin/tazobactam is an intermittent 30-minute infusion, potentially resulting in serum concentrations below minimum inhibitory concentration (MIC) for a prolonged period of time [11]. Numerous studies have investigated alternative dosing strategies that increase the drug’s fT>MIC, of which the extended or continuous infusion strategy (>3 hours) was an option [12–14], but no consistent conclusions were obtained. Therefore, a definitive recommendation is necessary because of its significant meaning for clinical practice. Though a meta-analysis comparing the two dosing strategies of piperacillin/tazobactam was done, the study had some limitations. Only two databases, PubMed and Scopus, were searched, articles published after January 2012 were not analyzed, and only one randomized controlled trial (RCT) was included in the meta-analysis [15]. Many new studies with better study design have been published since January 2012. Therefore, it is important and necessary to systematically investigate the clinical outcome differences between the two dosing strategies of piperacillin/tazobactam from those clinical trials in order to produce an evidence-based recommendation for clinical practice.

## Methods

### Literature search

Published articles were systematically searched (until April 30, 2014) from PubMed, Web of Science, ProQuest, Science Direct, Cochrane, two Chinese literature databases: [China National Knowledge Infrastructure (CNKI), Wan Fang Data] and related Interscience Conference on Antimicrobial Agents & Chemotherapy (ICAAC) and American College of Clinical Pharmacology （ACCP） conferences databases. References of the retrieved articles were also searched for additional studies. The following research pattern was utilized: (piperacillin/tazobactam) AND (extended OR continuous OR prolonged OR intermittent OR discontinuous OR short OR traditional OR conventional OR intermittent) AND (duration OR infusion OR administration OR interval OR dosing). No language restriction was applied to the search.

### Study selection

Articles reporting the comparative outcomes of patients treated with the two different dosing strategies of piperacillin/tazobactam were eligible for the meta-analysis, and the types of studies included were prospective study, retrospective study and RCT.

### Data extraction

Two reviewers (H.Y and Q.Y. Z) independently extracted relevant information for the meta-analysis. The extracted data included the characteristics of each study (author, study design, years, country), patient population (numbers of patients, type and etiology of infection), drug regimens, and clinical outcomes (clinical cure, mortality, bacteriologic cure, days in hospital, adverse events, cost) of the two groups in each study. Adverse events were directly described instead of statistical analysis considering few sample sizes.

### Quality assessment

Two authors (H.Y and Y.K.W) independently assessed the included studies for quality without blinding to journal or study authorship. Discrepancies were resolved by involvement of a third review author (C.Z) if required.

The quality of included RCT studies was assessed according to the criteria developed by the Cochrane risk of bias tool: random sequence generation, allocation concealment, blinding of participants and personnel, blinding of outcome assessment, incomplete outcome data, selective reporting and other bias. The quality of observational studies was assessed using the Newcastle-Ottawa Scales (NOS) [16].

### Statistical analysis

The meta-analysis was performed using Review Manager for Windows (version 5.2). Odds ratio (OR) and 95% confidence interval (CI) were calculated for each outcome. Statistical heterogeneity among studies was assessed by χ2 test (P <0.10 was defined to indicate significant heterogeneity) and I^2^ test. Mantel-Haenszel fixed effects model (FEM) was used when there was no significant heterogeneity between studies; otherwise, a random effects model was chosen. Adverse events were directly described instead of statistical analysis considering few sample sizes included. In order to evaluate the stability of results without estimation bias from individual study, especially considering relative small size studies being included [17–19], sensitivity analysis was performed by exclusion of each study one by one. This process of excluding one study at a time allowed for identification of any single article that may have a large influence on the final results. Publication bias was evaluated using the funnel plot method, of which funnel plot asymmetry was assessed by Egger’s linear regression test [20].

## Results

### Literature search

**Figure 1:**
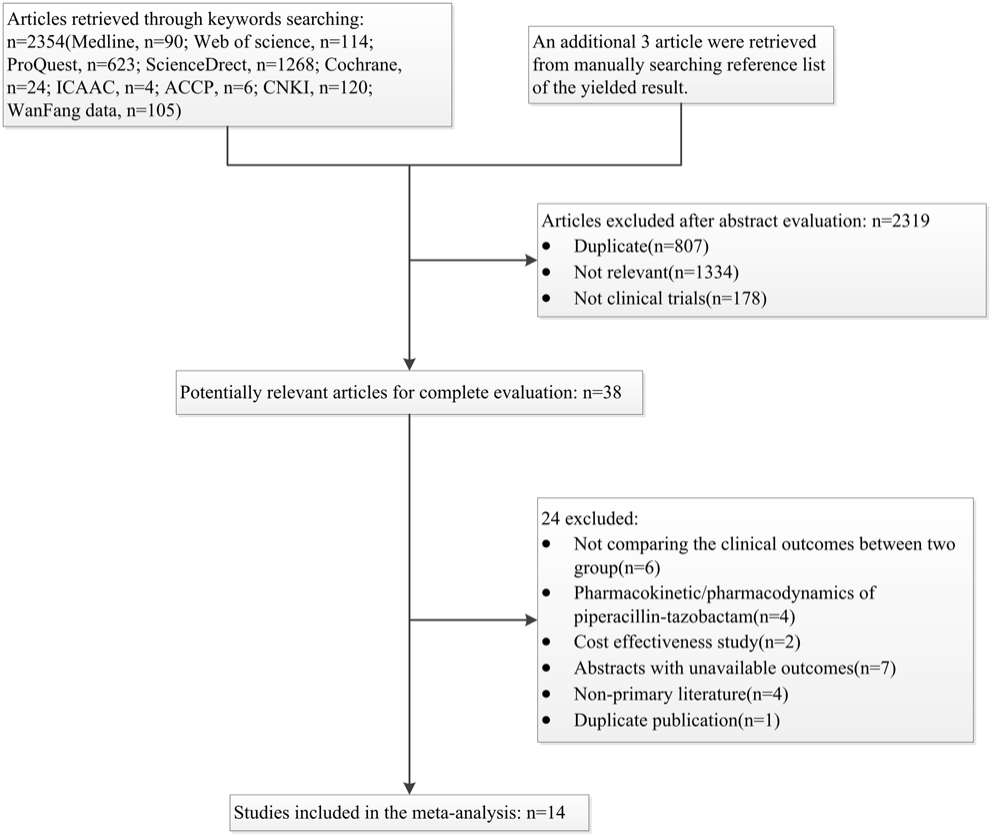
Flow chart depicting the selection process of studies included in the meta-analysis.

The search strategy yielded 2354 titles and abstracts. In addition, 3 articles were retrieved manually by searching from references lists. A total of 2319 articles were excluded after the review of abstracts, and 38 articles remained for full-text analysis. 24 articles were excluded after full-text review, leaving a total of 14 articles to be included in the analysis. The whole literature search process is summarized in Fig. 1.

### Study description

Characteristics of the eligible studies are presented in Table 1. This meta-analysis included fourteen studies, among which were two prospective studies [18], [21], seven retrospective studies [17], [24–26], [29–31] and five RCTs [19], [22], [27], [28], [32]. The patients of five of the included studies were persons who were admitted to the Intensive Care Unit (ICU) with severe infection, and the other nine studies included only non-ICU patients with moderate or severe infection. The infections of patients included pneumonia, sepsis, complicated intra-abdominal infection and ICU infections. The severity of underlying illness of the patients was evaluated using APACHE II, SAPS II or SOFA score and there was no statistically significant difference for their average values between two dosing groups in each study. In total, 1786 patients were included in the analysis and the sample sizes ranged from 16 to 359 in the identified studies. In the included studies, conventional intermittent infusion regimens were 2.25–4.5g over 20 or 30min three or four times daily. The extended infusion regimens lasted greater than 3 hours and the continuous infusion regimens lasted 24 hours with the doses ranging from 6.75 to 13.5g daily.

**Table 1:** The characteristics of included studies.

**Author, year, reference**	**Study design; years, country**	**No. of patients, infections**	**bacteria**	**CI or EI**	**II**	**Clinical cure**			**Mortality**			**Bacteriologic cure**		
						CI, n/N (%)	II, n/N (%)	P value	CI, n/N (%)	II, n/N (%)	P value	CI, n/N (%)	II, n/N (%)	P valve
**Grant,2002, [21]**	Prospective, open-lable;1999–2000, USA	98,NR	NR	9g q24h for HAP(n = 24), 13.5g q24h for nosocomial infections(n = 23)	3.375 q6h(n = 2),4.5g q8h(n = 49)	44/47(94)	42/51(82)	0.081	1/47(2.1)	5/51(9.8)	>0.5	25/28(89)	23/32(73)	0.092
**Buck,2005, [18]**	Prospective, open-lable;NR, Germany	24,CAP or HAP	NR	9g q12h(n = 12)^a^	4.5g q8h(n = 12)	8/12(67)	8/12(67)	>0.05	NR	NR	NR	NR	NR	NR
**Lau,2006, [22–23]**	RCT, open-lable;2002–2004,USA	167,cIAIs	Gram(-)/(+) bacteria	13.5g q24h(n = 130)^b^	3.375g over 30min q6h(n = 132)	70/81(86)	76/86(88)	0.817	1/130(0.8)	3/132(2.3)	>0.05	47/56(83.9)	51/58(87.9)	0.597
**Rafati,2006, [19]**	RCT;2003–2004,Iran	40,ICU septic	NR	8g daily over 24h g(n = 20)^c^	3g over 0.5h q6h(n = 20)	NR	NR	NR	5/20(25)	6/20(30)	0.72	NR	NR	NR
**Lodise,2007, [24]**	Retrospective cohort;2000–2004,USA	194,P. aeruginosa Infection	P. aeruginosa	3.375g over 4h,q8h(n = 102)^d^	3.375 over 30min, q4h or q6h(n = 92)	NR	NR	NR	5/41(12.2)	12/38(31.6)	0.04	NR	NR	NR
**Patel,2009, [25]**	Retrospective cohort;2006–2007,USA	129,Gram(-) infection	Gram(-) bacteria	3.375g over 4h,q8h(n = 70)	3.375 to 4.5g over 30min q6h or q8h(n = 59)	NR	NR	NR	4/70(5.7)	5/59(8.5)	0.54	NR	NR	NR
**Lorente,2009, [26]**	Retrospective cohort;2002–2007,Spain	83,VAP	Gram(-) bacteria	4.5g over 6h q6h(n = 37)^e^	4.5g over 30min q6h(n = 46)	33/37(89.2)	26/46(56.2)	0.001	8/37(21.6)	14/46(30.4)	0.46	NR	NR	NR
**Li,2010, [27]**	RCT;2006–2008,China	66,severe pneumonia	NR	4.5g 2ml/h bid(n = 28)	4.5g over 30min q8h(n = 31)	24/32(75.0)	17/34(50.0)	0.001	NR	NR	NR	11/32(34.4)	11/34(32.4)	<0.05
**Robort,2010, [17]**	Retrospective;2005,Australia	16,ICU	NR	13.5 continuous(n = 8)	4.5g over 20min q6h or q8h(n = 8)	8/8(100)	8/8(100)	NR	0/8(0)	0/8(0)	NR	NR	NR	NR
**Ye, 2011, [28]**	RCT;2009–2010,China	66,ICU	Gram(-) bacteria	4.5g over 3h q8h(n = 35)	4.5g over 30min q8h(n = 31)	24/35(68.6)	13/31(41.9)	<0.05	8/35(22.9)	8/31(25.8)	>0.05	15/35(42.8)	10/31(32.2)	>0.05
**Yost,2011, [29]**	Retrospective Cohort;2007–2010,USA	359,Pseudomonas aeruginosa infections	Pseudomonas aeruginosa	3.375g over 4h q8h(n = 186)	NR(n = 84)	NR	NR	NR	18/186(9.7)	17/84(20.2)	0.03	NR	NR	NR
**Pereira,2012, [30]**	Retrospective cohort;2006–2010,Portugal	346,ICU	NR	NR(n = 173)	t = 30min,dose NR(n = 173)	NR	NR	NR	49/173(28.3)	49/173(28.3)	1.0	NR	NR	NR
**Lee,2012, [31]**	Retrospective;2009–2011,USA	148,ICU	Gram(-) bacteria	3.375g over 4h q8h(n = 68)	2.25–4.5g over 30min q6h or q8h(n = 80)	NR	NR	NR	13/68(19)	30/80(38)	0.01	NR	NR	NR
**Lv,2013, [32]**	RCT;2012,China	50,HAP	Pseudomonas aeruginosa, e. coli, klebsiella pneumoniae	4.5g over 3h q6h(n = 25)	4.5g over 30min q6h(n = 25)	22/25(88)	20/25(80)		NR	NR	>0.05	NR	NR	NR

CI, Continuous infusion; EI, Extended infusion; II, Intermittent infusion; CAP, Community acquired pneumonia; HAP, Hospital acquired pneumonia; IAIs, Complicated intra-abdominal infection; ICU, Intensive care unit; VAP, Ventilator-associated pneumonia.

^a^ 2.5g single loading dose before starting continuous infusion.

^b^ A loading dose was administered before continuous infusion: 2.25g over 30min.

^c^ Loading dose was administered before continuous infusion: 2g.

^d^ Among patients with Acute Physiological and Chronic Health Evaluation-II score≥17.

^e^ A loading dose was administered before continuous infusion: 4.5g over 30min. A loading dose was administered before continuous infusion.

### Quality of included studies

Seven factors were used to evaluate the bias of the five included RCT studies according to the Cochrane risk of bias tool. Most factors for all studies showed low bias. However, the methods used to generate the allocation sequence in four studies were unclear. On the whole, the included RCTs in our study were of relatively high quality.

There were nine observational studies, including two prospective studies and seven retrospective studies. Eight factors were used to assess study quality according to NOS. The more factors the study met, the higher the quality of the study was. Except one study (Buck 2005) missed one indicator, the other seven studies were adequate in all criteria. The results showed that all observational studies were high quality.

### Clinical cure

**Figure 2:**
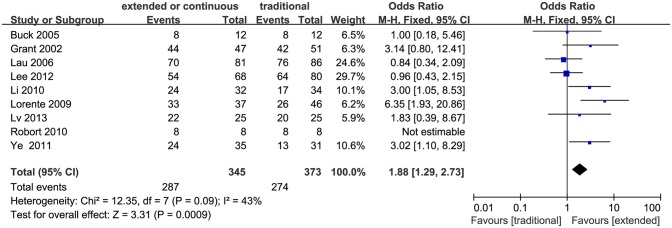
Forest plot depicting the odds ratios of clinical cure of patients receiving extended or continuous versus conventional intermittent infusion of piperacillin/tazobactam.

Clinical cure was defined as “cure” (the complete resolution of clinical signs and symptoms of infection, with no new signs or symptoms associated with the original infection) or “improvement” (the patient was not cured, but there was a resolution or a reduction of the majority of the clinical signs and symptoms of infection and no new or worsened signs associated with the original infection) in these studies. Nine studies including four RCTs, reported clinical cure rate [17], [18], [21], [22], [26–28], [31], [32]. Compared to the conventional intermittent infusion, the extended or continuous infusion had a significantly higher clinical cure rate (718 patients, OR 1.88, 95% CI 1.29–2.73, P = 0.0009; Fig. 2). No significant heterogeneity was found among all the studies (I^2^ = 43%, P = 0.09). The funnel plot did not show obvious asymmetry, and there was no publication bias presented by Egger’s test (P = 0.849).

### Mortality

**Figure 3:**
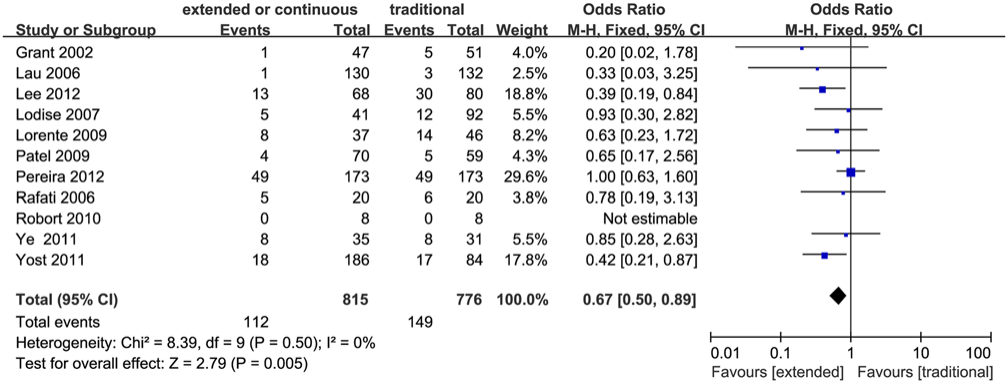
Forest plot depicting the odds ratios of mortality of patients receiving extended or continuous versus conventional intermittent infusion of piperacillin/tazobactam.

Eleven studies, including three RCTs, compared mortality rate between the extended or continuous infusion and the conventional intermittent infusion strategy [17], [19], [21], [22], [24–26], [28–31]. The extended or continuous infusion strategy was found having lower mortality rate compared to the conventional intermittent infusion (1591 patients, OR 0.67, 95% CI 0.50–0.89, P = 0.005; Fig. 3). No significant heterogeneity was found among the studies (I^2^ = 0%, P = 0.50). Obvious asymmetry was not found in the funnel plot. Egger’s test showed no publication bias, but the p value was 0.058, which indicated no statistically significant difference.

### Bacteriologic success

**Figure 4:**
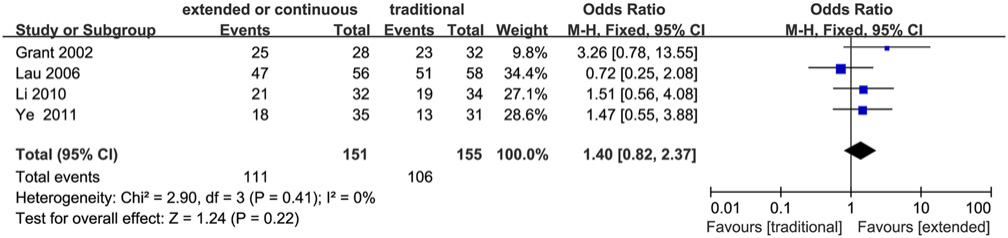
Forest plot depicting the odds ratios of bacteriologic success of patients receiving extended or continuous versus conventional intermittent infusion of piperacillin/tazobactam.

Bacteriological success was defined as success (“eradication” or “presumed eradication”) versus failure (“persistence” or “presumed persistence”). A total of four studies evaluated bacteriologic success, of which Li et al only reported overall success rates [27], and Grant et al [21], Lau et al [22], Ye et al [28] reported both overall and classified bacteriologic success rate for each type of bacteria. The average bacteriological success rate was 73.5% for the extended or continuous infusion group and 68.4% for the conventional intermittent infusion group, respectively. Bacteriological success rate showed no significant difference between the two infusion strategies (306 patients, OR 1.40, 95% CI 0.82–2.37, P = 0.22; Fig. 4).

### Adverse events

Adverse event data were few since piperacillin/tazobactam is rather well tolerated, and therefore, statistical analysis was not applied due to limited available data. Four studies described adverse events [17], [21], [22], [27] but two of them did not find any [17], [21]. In the RCT study by Lau et al, 22 of 130 patients (16.9%) in the continuous infusion group experienced adverse events versus 18 of 132 (13.6%) in the conventional intermittent group [22]. Six serious adverse events were found in the continuous group, including *Clostridium difficile* colitis, renal failure, confusion, tachycardia, and a tonic/clonic seizure, but none led to death. The incidence of adverse events was not significantly different between the two dosing groups. Another RCT study by Li et al reported 2 of 32 patients in the extended or continuous infusion group and 2 of 34 patients in the conventional intermittent group experienced adverse effects [27]. No serious adverse event was found in either group.

### Sensitivity analysis

The results of sensitivity analysis showed no substantial modification of the estimates after exclusion of individual study one by one.

## Discussion

This meta-analysis was performed to compare extended or continuous infusion with the conventional intermittent infusion approach of piperacillin/tazobactam. Our meta-analysis, including fourteen studies (two prospective studies, seven retrospective studies, and five RCTs), showed that the extended or continuous infusion strategy was associated with a higher clinical cure rate and lower mortality than the conventional intermittent approach. The bacteriological success rates and adverse events were not found to be significantly different between the two dosing approaches. These results ignored differences in severity of infection among different studies, but the average level of severity of infection between two dosing groups for each study was not significantly different.

Higher clinical cure rate for the extended infusion approach was found in our study, which is an important result, indicating the merit of using the extended or continuous infusion instead of the conventional intermittent infusion approach in clinical practice. The reason for which the extended or continuous infusion leads to increased clinical cure rate may be related to the increase in the time that the drug concentrations exceed the MIC since piperacillin/tazobactam is a time-dependent antimicrobial [1], [12–14], [17], [19], [24], [30], [33], [34]. However, Falagas et al concluded no difference in clinical cure rate between extended and conventional intermittent infusion strategy after their meta-analysis [15]. Possible reason may be related to limited numbers and few high quality studies included in their analysis (i.e. only one RCT incorporated). High quality researches with good study design are important and can avoid much of the bias of estimation [35]. As is well known, RCT is a study design of high quality, the best way to evaluate the clinical outcome, and can reduce bias to the maximum extent [36]. Five RCT studies were included in our analysis, avoiding possible bias to a large extent. Moreover, heterogeneity of the study is another main factor that may influence the final conclusion. FEM was used in our analysis since there was no significant statistical heterogeneity present among included studies (Fig. 2). If significant heterogeneity did exist, a random effect model would have been used instead, similar to the work of Falagas et al. Compared to the random-effects model, the fixed-effects model is more sensitive and accurate [37], [38]. Sometimes, an opposite result could even be obtained using the random effect model compared to the fixed effect model [39]. Sensitivity analysis is used to measure the stability of results, especially when heterogeneity exists. It is a good way to find the source of heterogeneity and eliminate it. There was one small size study included in the analysis [18], but it did not modify the conclusion of the study when excluded during the sensitivity analyses.

Our meta-analysis suggested that extended or continuous infusion of piperacillin/tazobactam resulted in significantly lower mortality rate compared to the conventional intermittent infusion. No publication bias was discovered and the sensitivity analysis showed no substantial modification in this meta-analysis, but the Egger’s test result did not reveal obvious statistical significance.

The bacteriologic success rate was not found to be statistically different in this meta-analysis. The β-lactam antibiotics exhibit their bactericidal effects by inhibiting enzymes involved in cell wall synthesis [40]. However, bacteriologic success rate is not as good as other endpoints since it is easily influenced by many factors, of which some are not measured routinely, for example, the specimen storage conditions. In most instances, the end point is imputed (i.e. clinical success without resampling from the infection site equals microbiological success). There were four included studies that reported bacteriologic cure, of which two were from the United States [21], [22] and two were from China [27], [28]. The bacteriologic success rate was about 80% in the United States, which was relatively high, compared to about 50% in China. Low bacteriologic success rate may be attributed to high resistance rates due to overuse in some areas of China [41], [42]. No matter what reasons led to a lower bacteriologic success rate, a better dosing strategy is linked to increased bacteriologic success rate and more rational use of antimicrobials.

Drug-related adverse effects were mild and reported in similar numbers in both dosing strategy in all studies. This was powerful evidence against the traditional concept that an extended or continuous infusion could further induce toxicity reactions due to the high drug concentration lingering for a long-time within tissues [43]. However, more well-designed trials are needed to clarify this issue.

Eight different studies, including one RCT, provided inconsistent results towards the question that whether the extended or continuous infusion approach of piperacillin/tazobactam could reduce the length of hospital stay [21], [24–26], [29–32]. Grant et al reported that days of therapy were similar with both treatment groups (7.3 ± 4.8 days for continuous infusion versus 8.7 ± 7.1 days for conventional intermittent infusion, P = 0.26) [21]. This finding was also found by Patel et al [25], Lorente et al [26], Yost et al [29] and Pereira et al [30]. However, Lodise et al found that the median duration of hospital stay was significantly shorter in patients who received extended infusion therapy compared to conventional intermittent therapy (21 days versus 38 days, P = 0.02) [24]. Lee et al [31] and Lv et al [32] also found a similar result when comparing the mean duration of therapy.

MIC ranges and pathogens are very important factors for consideration of their influence on patient outcomes. Unfortunately, the studies included in the meta-analysis did not report data based on the source of infection. However, the evaluation indicators used in this study were clinical signs and symptoms, which were consistent with the types of pathogens and MIC ranges between two dosing groups, reducing the influence to the maximum extent.

Budget restrictions have put most institutions under pressure to curtail pharmacy costs. Except studies by Grant et al [21] and Heinrich et al [44], no economic advantage was observed for the extended or continuous infusion strategy in other studies. A lower total daily dose may be required for the extended infusion in order to achieve a similar drug concentration [45],which is the link to a decreased cost. Nevertheless, based on the current available evidence, we cannot make the conclusion that the extended or continuous infusion of piperacillin/tazobactam has any economic advantage.

All studies that we analyzed were of high quality, including RCTs and observational studies. Therefore, our conclusions were relatively reliable, but there were still multiple confounding factors. Small sample trials might bring bias. Additionally, disease status and drug doses were not the same in all studies, which could influence the clinical outcomes. Also, information regarding concurrent medications was not given in the studies analyzed. Therefore, drug-drug interactions were unknown and could not be considered during our evaluation.

In conclusion, evidences demonstrated that the extended or continuous infusion of piperacillin/tazobactam led to a higher clinical cure rate and a lower mortality rate than the conventional intermittent strategy. Therefore, this alternative infusion strategy could be recommended in clinical practice. Further data on the impact on adverse effects and economic budget should be generated for a better understanding of the extended or continuous infusion strategy.

## Supporting Information

S1 PRISMA Checklist PRISMA checklist of this meta-analysis. (DOC)

S1 Fig PRISMA flow diagram. (DOC)
