# Supplementary material for: Clinical Outcomes with Alternative Dosing Strategies for Piperacillin/Tazobactam: A Systematic Review and Meta-Analysis
Source: PLoS One. 2015 Jan 9;10(1):e0116769. doi: 10.1371/journal.pone.0116769 (PMC4289069; doi:10.1371/journal.pone.0116769)
Supplement: S1 Fig — (DOC) [file pone.0116769.s002.doc]

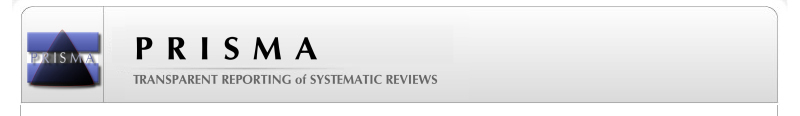
**PRISMA 2009 Flow Diagram**

**Screening**

**Included**

**Eligibility**

**Identification**

Records identified through database searching
(n =2354 )

Additional records identified through other sources
(n = 3 )

Records after duplicates removed
(n =807 )

Records screened
(n =1550 )

Records excluded
(n =1512 )

Full-text articles assessed for eligibility
(n = 38 )

Full-text articles excluded, with reasons
(n =24 )

Studies included in qualitative synthesis
(n =14 )

Studies included in quantitative synthesis (meta-analysis)
(n = 14 )
